# A Case of Idiopathic Cold Agglutinin Hemolytic Anemia Successfully Treated With Steroids

**DOI:** 10.7759/cureus.23172

**Published:** 2022-03-15

**Authors:** Asim Haider, Fareeha Alavi, Ayesha Siddiqa, Muhammad Owais, Muzammil Khan

**Affiliations:** 1 Internal Medicine, BronxCare Health System, Bronx, USA; 2 Infectious Disease, NewYork-Presbyterian Queens, Queens, USA; 3 Respiratory Medicine, Rush University Medical Center, Chicago, USA; 4 Internal Medicine, Stony Brook University Hospital, Brookhaven, USA

**Keywords:** autoantibodies, direct coombs test, anemia, cold agglutinin disease, immune hemolytic anemia

## Abstract

Cold agglutinin disease (CAD) is a type of hemolytic anemia in which cold agglutinins can cause agglutination of red blood cells in cold parts of the body and hemolytic anemia. Cold agglutinin-mediated hemolytic anemia can occur in the setting of an underlying viral infection, autoimmune disorder, or lymphoid malignancy, referred to as a secondary cold agglutinin syndrome, or without one of these underlying disorders, referred to as primary CAD (also known as idiopathic CAD). We present a case of a 71-year-old female with hemolytic anemia due to primary CAD. The secondary causes of CAD, including infections, autoimmune disorders, and malignancy, were ruled out. She was successfully treated with prednisone.

## Introduction

Cold agglutinin disease (CAD) is characterized by the agglutination of red blood cells (RBCs) when exposed to cold (usually in the extremities) and hemolytic anemia. Cold-sensitive antibodies are usually classified into three major classes: (a) Cold agglutinins are the immunoglobulins that attach to the antigens of RBCs causing them to agglutinate and hemolyze. This hemolysis is usually extravascular, leading to anemia without significant hemoglobinuria. (b) Donath-Landsteiner antibodies attach to RBC antigens at a cold temperature similar to cold agglutinins, but, in contrast to cold agglutinins, these antibodies fix complement leading to intravascular hemolysis and hence cause hemoglobinemia and hemoglobinuria. This is called paroxysmal cold hemoglobinuria (PCH). (c) Cryoglobulins are the third type of cold-sensitive antibodies that form immune complexes in the cold temperature and cause systemic vasculitis. These antibodies usually do not react with RBCs. Cold agglutinin-associated autoimmune hemolytic anemia (AIHA) can be primary (idiopathic) or secondary (due to viral infections, lymphoproliferative disorder, or autoimmune disorder) [1]. 

## Case presentation

A 71-year-old Hispanic female presented to the emergency department with the complaint of generalized weakness for three days. The patient was referred to the emergency department by her primary care physician due to low hemoglobin of 5.1 g/dL on routine blood work. She denied any chest pain, palpitations, shortness of breath, dizziness, headache, rectal bleeding, dark stool, melena, hematochezia, hematemesis, epistaxis, hemoptysis, vaginal bleeding, nonsteroidal anti-inflammatory drugs (NSAIDs) consumption, hematuria, or easy bruising. The patient had medical comorbidities of hypertension, diabetes mellitus, hyperlipidemia, and obstructive sleep apnea, for which she was on lisinopril, metformin, and atorvastatin. The patient had a colonoscopy one year ago, which was unremarkable. 

Upon presentation, the patient had a temperature of 98.6°F, blood pressure of 137/67 mmHg, a pulse of 83 beats per minute, and oxygen saturation of 100% on room air. On physical examination, the patient was alert and comfortable. Heart and lung sounds were unremarkable. The abdomen was soft and nontender. Initial laboratory findings were significant for severe macrocytic hemolytic anemia with hemoglobin of 5.1 g/dL, mean corpuscular volume (MCV) of 120 fL, and indirect hyperbilirubinemia of 3.3 mg/dL. The patient was also found to have a high reticulocyte count, high lactate dehydrogenase (LDH), and low haptoglobin. A direct Coombs test was sent given hemolytic anemia, which was positive for IgG-C3bC4d antibodies (Table 1). The fecal test was negative for any occult blood. Her cold agglutinin antibody testing revealed elevated antibody titers (1:610 at 4°C). 

**Table 1:** Laboratory findings for the patient

Parameter	Value	Reference Range
Hemoglobin (g/dL)	5.1	12.0-16.0
White blood cell count (x10^3^/µL)	6.2	4.8-10.8
Platelet (x10^3^/µL)	180	150-400
Mean corpuscular volume (fL)	120	80-96
Reticulocyte %	18.7	0.5%-1.5%
Sodium, serum (mEq/L)	140	135-145
Potassium, serum (mEq/L)	4.0	3.5-5.0
Blood urea nitrogen, serum (mg L)	12.0	6.0-20.0
Creatinine, serum (mg/dL)	0.8	0.5-1.5
Alanine aminotransferase, serum (U/L)	18	<5-40
Aspartate aminotransferase, serum (U/L)	29	9-48
Bilirubin, serum total (mg/dL)	3.3	0.2-1.1
Bilirubin, serum conjugated (mg/dL)	1.0	0.0-0.3
Lactate dehydrogenase, serum (U/L)	459	110-210
Haptoglobin, serum (mg/dL)	<10	30.0-200
Direct Coombs test	Positive IgG-C3bC4d	Negative
Serum folate (ng/mL)	7.9	3.0-19.9
Serum iron (µg/dL)	71	65-175
Unsaturated iron binding capacity (µg/dL)	170	113-346
Serum vitamin B12 (pg/mL)	835	243-894
Antinuclear antibody	Negative	Negative
Epstein-Barr virus immunoglobulin M (IgM) (U/mL)	<36	<36
Blood glucose level, serum (mg/dL)	77	70-120
Hepatitis C virus antibody	Negative	Negative
Hepatitis B surface antibody	Positive	Positive: immune negative: not immune
Hepatitis B surface antigen	Negative	Negative
Mycoplasma pneumoniae IgM (U/mL)	406	<770
Serum immunoglobulin A level (mg/dL)	109	70-320
Serum immunoglobulin G level (mg/dL)	683	600-1540
Serum immunoglobulin M level (mg/dL)	53	50-300
Serum C3 complement (mg/dL)	119	90-150
Serum C4 complement (mg/dL)	3.0	16.0-47.0
Prothrombin time	14.4	9.9-13.3 seconds
Partial thromboplastin time	31.4	27.2-39.6 seconds

**Figure 1:**
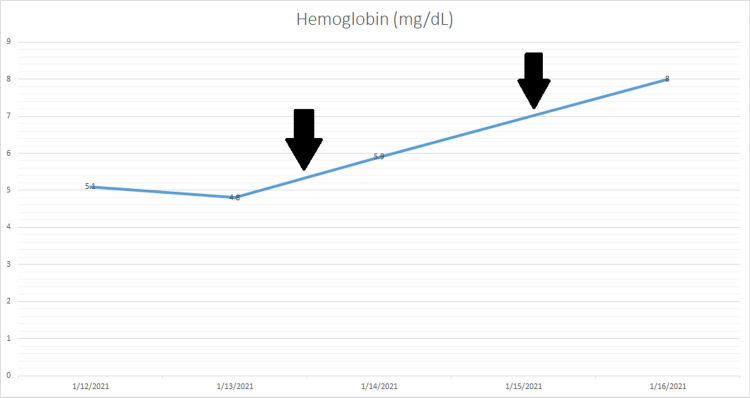
Graph showing trend of hemoglobin following packed red blood cells transfusion (black arrows) over the period of admission

The workup for secondary CAD disease was negative. Her serum *Mycoplasma pneumoniae* immunoglobulin M (IgM) and Epstein-Barr virus IgM were negative. Serum antinuclear antibodies (ANAs) were also reported to be negative. A computerized tomography scan of the chest and abdomen with contrast material was negative for any lymphadenopathy or mass to suspect lymphoma or malignancy. The patient was diagnosed with primary CAD; she was started on prednisone 40 mg twice daily. Her hemoglobin improved after she received two units of appropriately warmed packed RBCs transfusion. Repeat complete blood count showed improvement in hemoglobin to 8.0 g/dL (Figure 1).

With improvement in her clinical status, she was discharged with outpatient hematology follow-up. She continues to follow-up in the clinic. Her prednisone dose has been gradually tapered down to 5 mg/day with stable hemoglobin counts.

## Discussion

Primary CAD is a rare disease. Berentsen et al. reported an incidence of one per million in a study done in Norway [2]. The median age of diagnosis is usually 60s to 70s. The surface of RBCs contains various antigenic epitopes. The antibodies (typically immunoglobulin M (IgM)) bind to these antigens in areas of the body with low temperature (usually extremities, especially when the ambient temperature is low) [3,4]. IgM then activates the complement system (classical pathway), which in turn stimulates the reticuloendothelial system leading to hemolysis [5]. The hemolysis in CAD is extravascular and is medicated by the complement system [6]. If RBCs are not phagocytosed by the reticuloendothelial system, IgM dissociated upon warming, but the complement mediators remain attached (especially C3d), which can be detected using the Coombs test [7]. A positive Coombs test is one of the initial tests to suggest CAD.

The clinical features of CAD vary from asymptomatic cases to severe anemia. Many individuals have circulating cold agglutinins in the blood but are unaware of this unless they are exposed to cold temperatures. There have been cases of severe hemolysis leading to multiorgan failure in patients with cold agglutinins who were exposed to therapeutic hypothermia (e.g., for cardiac surgery) [8]. The severity of hemolysis can range from compensated hemolysis without anemia to severe hemolytic anemia requiring transfusion [1]. Median hemoglobin levels are about 9 to 10 g/dL [3]. Cold-induced symptoms in the extremities (e.g., cyanosis, livedo reticularis, ulceration, Raynaud phenomenon, or discomfort on swallowing cold food) are extremely common in CAD [9].

The typical diagnostic approach starts with a complete blood count (CBC) and a peripheral blood smear review. The CBC may or may not show anemia depending upon the degree of hemolysis. Usually, the reticulocyte count is increased (could be normal if the hemolysis was recent or if there is an underlying bone marrow disorder). The lactate dehydrogenase (LDH) and bilirubin are increased, and the haptoglobin is decreased or absent. The direct Coombs test is positive for the C3b complement, while C3 and C4 were usually consumed [3]. The threshold for cold agglutinin titers is usually considered to be 64, but most experts consider titers above 512 to be diagnostic [5]. The specimen collected for cold agglutinin testing must be maintained at 37°C to 40°C until the formation and retraction of the clot; otherwise, the cold agglutinin precipitates and may be removed during the preparation of the sample. The following criteria are generally accepted for the diagnosis of CAD: (a) evidence of hemolysis (e.g., high reticulocyte count, high LDH, high indirect bilirubin, low haptoglobin), (b) positive direct antiglobulin (Coombs) test for C3d only (or, in the minority, C3d plus IgG), and (c) cold agglutinin titer of ≥64 at 4°C [10]. 

Secondary causes should be evaluated to rule out any underlying pathology responsible for CAD. If respiratory symptoms are present, testing for an infectious disorder (e.g., infectious mononucleosis, mycoplasma) is appropriate. Testing for an autoimmune disorder is recommended in those with features of autoimmune disorders (e.g., arthralgias, rash, or cytopenias). Testing for a lymphoproliferative disorder (e.g., non-Hodgkin lymphoma or Waldenstrom macroglobulinemia) is done if there is weight loss, hepatosplenomegaly, lymphocytosis, or cytopenias. Most cold agglutinins associated with infections or autoimmune disorders are likely to be polyclonal and will resolve spontaneously with a resolution of the infection (which may include antibiotic therapy) or treatment of the autoimmune disorder. Cold agglutinins associated with lymphoid are likely to be monoclonal and will not resolve spontaneously or respond to glucocorticoids or splenectomy. If these individuals have significant hemolysis, treatment will require therapy to eradicate the clone of cells producing the cold agglutinin.

In contrast to warm AIHA, CAD is not responsive to splenectomy or glucocorticoids, although cases have been successfully treated with steroids [11,12]. The cases which respond to the steroid therapy usually have one of the following features: (a) low antibody titers, (b) the antibodies have a higher thermal range causing some degrees of warm hemolysis, (c) mixed warm and cold AIHA, and (d) predominant antibodies are IgG cold-reacting antibodies [3,13]. An antibody titer less than 1,000 is usually considered to be a low titer [14]. Our patient had a titer of 1:610 and responded well to steroids. 

## Conclusions

CAD is an uncommon cause of hemolytic anemia. It can be primary (idiopathic) or secondary to other etiologies, including infectious disorder (e.g., infectious mononucleosis, mycoplasma), autoimmune disorders, lymphoproliferative disorder, or lymphoid malignancy. The secondary causes of CAD should be ruled out. All patients should avoid cold exposures until the underlying cause of CAD has resolved or been eliminated. Primary CAD is usually resistant to treatment with glucocorticoids, but there have been a few cases of CAD successfully treated with glucocorticoids.
